# Adenosine Deaminase 2 Deficiency (DADA2): A Crosstalk Between Innate and Adaptive Immunity

**DOI:** 10.3389/fimmu.2022.935957

**Published:** 2022-07-11

**Authors:** Sara Signa, Arinna Bertoni, Federica Penco, Roberta Caorsi, Alessia Cafaro, Giuliana Cangemi, Stefano Volpi, Marco Gattorno, Francesca Schena

**Affiliations:** ^1^ Center for Autoinflammatory Diseases and Immunodeficiencies, Istituto di Ricovero e cura a carattere scientifico (IRCCS) Istituto Giannina Gaslini, Genoa, Italy; ^2^ Department of Neuroscience, Rehabilitation, Ophthalmology, Genetics and Maternal-Child Sciences (DINOGMI), University of Genoa, Genoa, Italy; ^3^ Chromatography and Mass Spectrometry Section, Central Laboratory of Analysis, Istituto di Ricovero e cura a carattere scientifico (IRCCS) Istituto Giannina Gaslini, Genoa, Italy

**Keywords:** adenosine deaminase 2 (ADA 2), DADA2, innate immunity, adaptive immunity, TNF-α, interferon

## Abstract

Deficiency of Adenosine deaminase 2 (DADA2) is a monogenic autoinflammatory disorder presenting with a broad spectrum of clinical manifestations, including immunodeficiency, vasculopathy and hematologic disease. Biallelic mutations in ADA2 gene have been associated with a decreased ADA2 activity, leading to reduction in deamination of adenosine and deoxyadenosine into inosine and deoxyinosine and subsequent accumulation of extracellular adenosine. In the early reports, the pivotal role of innate immunity in DADA2 pathogenic mechanism has been underlined, showing a skewed polarization from the M2 macrophage subtype to the proinflammatory M1 subtype, with an increased production of inflammatory cytokines such as TNF-α. Subsequently, a dysregulation of NETosis, triggered by the excess of extracellular Adenosine, has been implicated in the pathogenesis of DADA2. In the last few years, evidence is piling up that adaptive immunity is profoundly altered in DADA2 patients, encompassing both T and B branches, with a disrupted homeostasis in T-cell subsets and a B-cell skewing defect. Type I/type II IFN pathway upregulation has been proposed as a possible core signature in DADA2 T cells and monocytes but also an increased IFN-β secretion directly from endothelial cells has been described. So far, a unifying clear pathophysiological explanation for the coexistence of systemic inflammation, immunedysregulation and hematological defects is lacking. In this review, we will explore thoroughly the latest understanding regarding DADA2 pathophysiological process, with a particular focus on dysregulation of both innate and adaptive immunity and their interacting role in the development of the disease.

## Introduction

Since the first description of Adenosine Deaminase 2 deficiency (DADA2) in 2014 (1, 2), we have witnessed an exponential expansion of its possible clinical phenotypes. The clinical manifestations range from the initial descriptions of systemic inflammation with fever, early onset strokes and vasculopathy (3–10) to immune dysregulation (hypogammaglobulinemia, lymphoproliferation, increased infection rate) (5, 11–13) and hematological abnormalities (PRCA, bone marrow (BM) failure, cytopenias) (5, 6, 13–17), with several reports of different features overlapping (12, 18).

**Figure 1:**
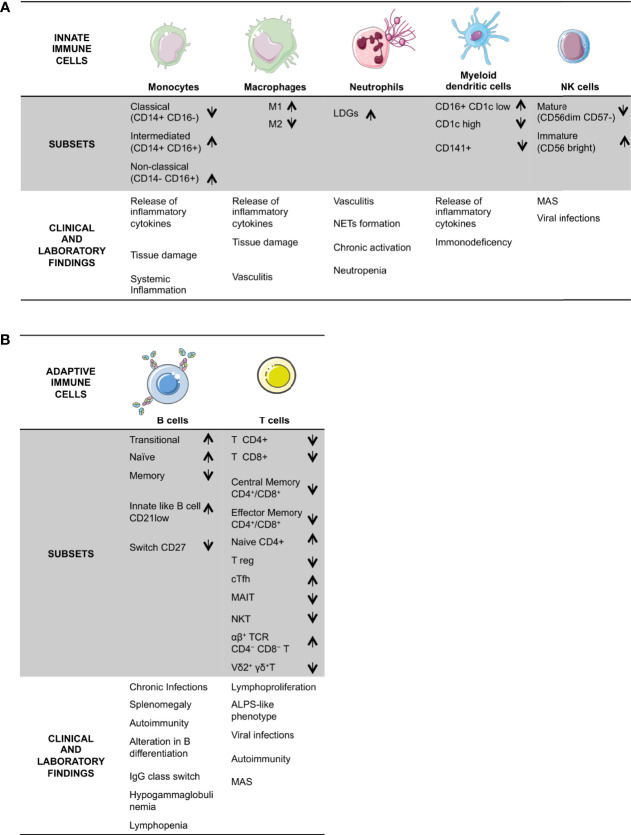
Schematic overview of DADA2 alterations in immune cells and the described related clinical signs and laboratory findings. DADA2 is associated with a wide variety of clinical manifestations. Here is a summary of clinical and immunological features, grouped in the two branches of the immune system; innate **(A)** and adaptive **(B)**. LDG, Low Density Granulocytes; Treg, regulatory T cells; cTfh, circulating Tfh; MAIT, mucosal-associated invariant T; MAS, macrophage activation syndrome; ALPS; Autoimmune Lymphoproliferative Syndrome.

The polymorphic DADA2 clinical presentation reflects the ADA2 defect’s deep impact on different immune response branches (**Figure 1**), even if the possible presence of different clinical manifestations in patients with the same mutation (3, 8, 19, 20) advocates for further factors influencing the clinical phenotype. Recently a pivotal work unraveled in DADA2 an association of partial loss of ADA2 activity with the vasculitic phenotype and predominant missense mutations, whereas complete absent activity was observed in hematologic disease (13).

ADA2 is a dimeric enzyme that, similarly to its ADA1 isoform, catalyzes the deamination of adenosine and 2’-deoxyadenosine into inosine and deoxyinosine respectively (21–23). ADA2 exhibits not only an enzymatic activity, but also a growth factor role (22, 23) and it is required for a proper monocytes-T cells interaction (23, 24).

Notably, the determination of ADA2 activity is essential in the current diagnosis of DADA2, since pathogenic variants may not be detected by conventional genetic testing and may require the incorporation of additional diagnostic methods (25, 26). Few methods are currently available for a reliable ADA2 enzyme testing (1, 13, 27–29), some of them based on chromatographyc assays for quantification of adenosine and inosine (1, 7, 9, 30).

Spectrophotometric methods, with less specificity, have been reported (31–34). Recently, a novel enzymatic assay based on liquid chromatography-tandem mass spectrometry, that allows the accurate determination of the ADA2 activity starting from very small amounts of plasma spotted on DBS has been described (35).

## The Impact of ADA2 Deficiency on the Cross Talk Between Innate and Adaptive Immunity

ADA2 protein is highly expressed in immune cells, particularly in myeloid ones (23, 24, 36), from which is actively secreted (23) and binds several immune cell lineages, such as monocytes but also B cells, neutrophils and NK cell (24). Therefore, the impact of ADA2 deficiency on different branches of the immune system is not unexpected.

**Figure 2:**
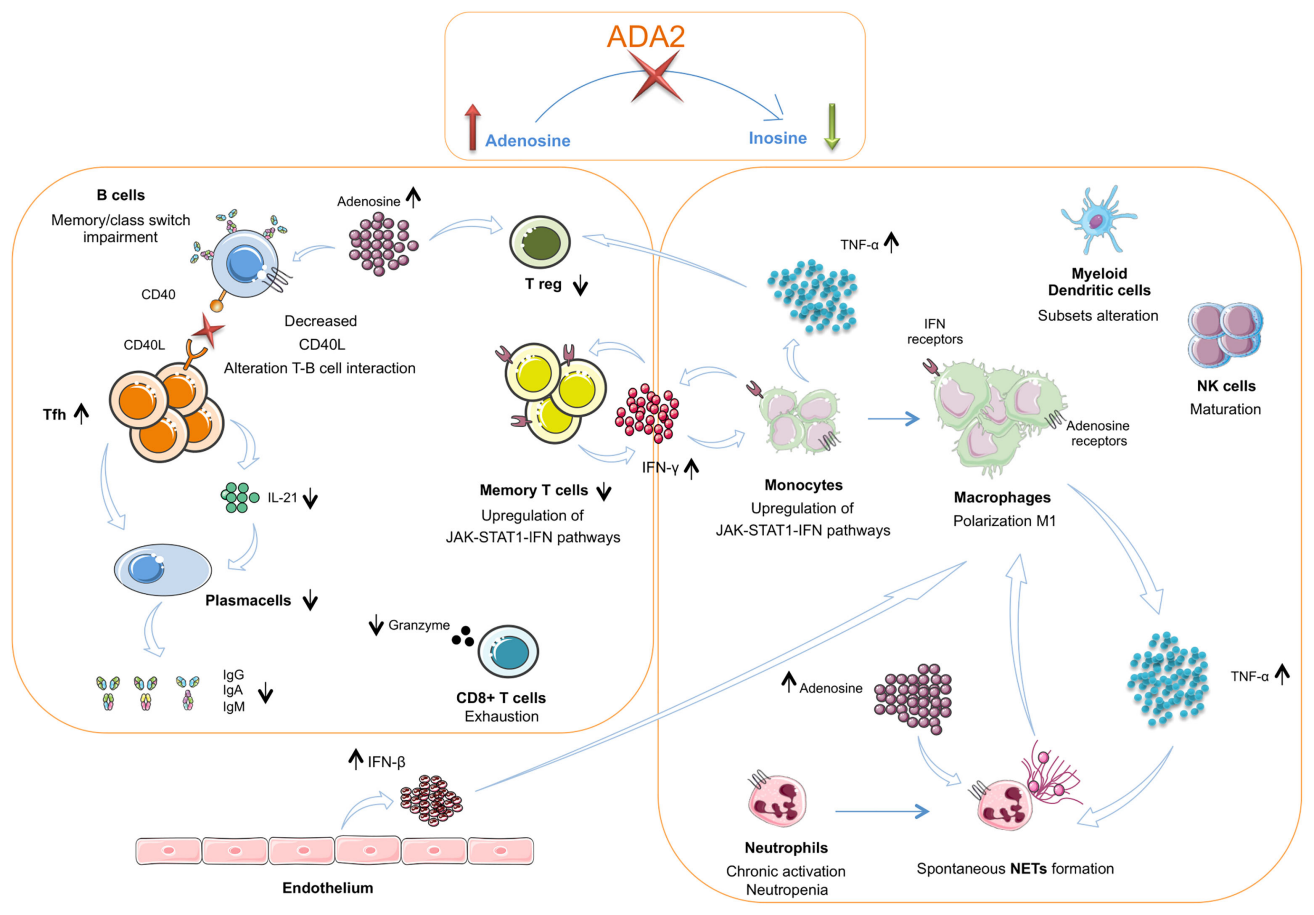
The interplay between innate and adaptive immune cells in DADA2. The insufficient or absent ADA2 enzymatic activity leads to a reduction of adenosine conversion into inosine and to an accumulation of adenosine in the extracellular space. In the innate immune system this leads to a chronic neutrophils activation and NETosis dysregulation with spontaneous NETs formation. Macrophages show a polarization towards the M1 subtype with consequent hypersecretion of inflammatory cytokines, especially TNF-α Dysregulation of NETosis and IFN-β secreted in large amount by endothelium also contribute to the M1 polarization. TNF-α acts on neutrophils contributing to maintaining their chronic activation state. CD56bright immature NK cells are increased. Dendritic cells present an altered subsets distribution. TNF-α, together with adenosine, acts also on Treg cells, that result diminished. In the T cell compartment, Tfh cells show an increased frequency but an impaired secretion of IL-21, crucial for B-cell function. Furthermore, T cells present a reduced proportion of the memory subsets and an alteration of CD40L expression. This defect is associated with an impaired B cell proliferation, differentiation and switching with consequent lower levels of immunoglobulins. CD8+ T cells show an exhausted phenotype and produce low amounts of granzyme A. Both T cells and monocytes show an upregulation of JAK – STAT1 – IFN pathways. In monocytes this activation contributes to the M1 macrophage polarization. TNF-α, Tumor necrosis factor α; IFN β, Interferon β; NETs, Neutrophils extracellular traps.

The present review aims to clarify innate and adaptive immunity’s main peculiarities identified in DADA2 patients, and their possible interconnections leading to DADA2 polymorphic manifestations (**Figure 2**).

### Monocytes and Macrophages, the Drivers of Inflammatory Cytokines

Since the original reports, the pivotal role of monocytes and macrophages inducing the inflammatory response in DADA2 has been outlined. Zhou and colleagues (1) showed a skewed *in vitro* monocyte differentiation, with increased proinflammatory M1 macrophages and relatively decreased anti-inflammatory M2 macrophages subset in DADA2 patients. This monocyte/macrophage polarization is supposed to induce a release of proinflammatory cytokines, leading to downstream endothelial disruption (1, 37). Notably, the lentiviral correction of defective ADA2 enzymatic activity with ADA2 reconstitution in patients macrophages is able to restrain inflammation (36, 38).

Despite initial reports not being univocal, a preferential binding of ADA2 to CD16+ monocytes instead to the “classical” CD16- monocytes is reported (24). An in-depth immune-phenotyping analysis of prevalently untreated DADA2 patients showed significantly reduced proportions of classical monocytes (CD14+ CD16-) with an increased proportions of intermediated (CD14+ CD16+) and non-classical (CD14- CD16+) monocytes (12). This finding has been confirmed by Watanabe et al (39), who analyzed CD14+ monocytes subpopulations through single cell RNA sequencing, identifying a higher frequency of nonclassical monocytes and an up-regulation of M1 macrophage markers compared with healthy donors. Indeed, non-classical monocytes are a prominent source of TNF-α compared with classical monocytes (40–42), and these findings are in line with the pathogenic role of TNF-α. Interestingly, no differences were found comparing monocyte transcriptomes of patients with a hematologic phenotype versus a nonhematologic phenotype, suggesting that the explanation for the BM failure observed in DADA2 is to be sought elsewhere (39, 42).

A possible pivotal role in DADA2 pathogenesis has been recently attributed to a dysregulation of the IFN type I and II axis. An upregulation of several pathways involved in the immune response, including IFNα/β and IFNγ signaling, was evident in all monocyte subsets (39). This observation was in line with the identification of elevated plasma levels of IFNγ. Similar findings were reported in other studies profiling transcriptome and proteome analysis on whole blood samples or gene expression on DADA2 PBMCs (37, 43). The authors suggested that elevated IFNγ may activate cell signaling, leading to monocytes differentiation into M1 macrophages and release of TNFα (39). Interestingly, Yap and colleagues (12) identified a twofold higher level of expression of CD169/SIGLEC1 on all subsets of ADA2-deficient monocytes compared to controls. CD169/SIGLEC1 is a cell adhesion protein induced by type I IFN-signaling and a potential myeloid cell-specific biomarker for an overt type I IFN response on monocytes (44). This finding is in line with reported evidence of upregulated type I IFN signaling in DADA2 with elevation of IFN score (12, 37, 43–48).

Finally, an upregulation of STAT1 phosphorylation in DADA2 monocytes after stimulation with IFNα and IFNγ was recently shown (37) confirming similar findings regarding STAT1 central role in altered DADA2 immune response (43, 49).

Together with IFN type I, the latter study also identified a clear NFKB signature in the whole blood of untreated patients, with normalization of both findings after anti-TNF treatment (37).

### Natural Killer (NK) Cells and Dendritic Cells (DCs): The Producers of Interferons in DADA2?

NK cells are mainly involved in antiviral and antitumor response (50–52). ADA2 can bind NK cells, so NK is a target cell to ADA2 (23). After the initial reports showing a reduction in NK numbers (24) in DADA2 patients a consensual reduction of mature CD56 dim CD57- NK cells, displaying high cytotoxicity and low cytokine production, was found (3, 12). An increased proportion of immature CD56 bright NK cells, known as NK regulatory (53), was also identified (12). These later cells are characterized by low cytotoxicity and high cytokine production, including TNF-α. It would be fascinating to investigate the functional role of this subset, particularly to understand the possible involvement in some peculiar clinical features described in DADA2, such as recurrent viral infections (12, 27, 54, 55) and macrophage activation syndrome (MAS) (1, 5, 56) (**Figure 1**).

The role of DCs in DADA2 patients has not yet been investigated thoroughly. ADA2 is highly expressed in DCs^1^, which secrete ADA2 continuously (23). The in-depth immune phenotyping of DCs (12) showed that ADA2-deficiency does not impact on overall numbers and proportions of DCs. However, the distribution of myeloid DCs (mDCs) subsets was altered, with significant reduction in the proportion of CD1c-hi and CD141+ subsets. Conversely, a significantly increased proportion of CD16+CD1c lo/− mDCs was found in DADA2 patients compared with healthy controls. These DCs, or non-classical like monocytes, have potent T cell stimulatory capacity and produce proinflammatory cytokines upon TLR stimulation by TNF-α, IL6, IL12 (12). Thus, these cells may play a role in inducing and maintaining the inflammatory state observed in DADA2. In addition, reduced proportions of mDCs may contribute to the overall defect in T and B cell immune responses observed *in vivo* in DADA2 patients. It could be speculated that the DCs activated by exogenous triggers and the plasmocytoid DCs (pDCs) stimulated by self-antigens may secrete huge amounts of type I IFN. In turn, this process can act as an autocrine and paracrine stimulus on pDCs, monocytes and other cells extending the inflammatory cascade. So far, functional studies supporting this hypothesis are missing.

### Neutrophils and Interplay With Innate Immune Cells

Neutrophils generate and release adenosine at sites of inflammation (57–59) and express receptors for adenosine (24), which may play several effects on their function (57). The zebrafish DADA2 model presented neutropenia (1), which may be part of the clinical phenotype (7, 60, 61). In DADA2 patients a marked upregulation of neutrophil expressed gene transcripts, a chronic activation of neutrophils and an increased expression of myeloperoxidase in PBMCs, possibly leading to endothelial damage (46), were demonstrated. Carmona-Rivera and colleagues (62) described the pathogenic role of neutrophils and the possible involvement of NETosis in the induction of tumor necrosis factor (TNF)-α release from macrophages (**Figure 2**).

DADA2 patients present a higher proportion of low-density granulocytes (LDGs), which have been partly implicated in the pathogenesis of vasculitis through the formation of neutrophil extracellular traps (NETs) (63–65).

The increase of circulating LDGs, prone to spontaneous NETs formation, was observed in DADA2 patients in active disease, with a significant reduction in disease remission after anti–TNF therapy. Adenosine was demonstrated to trigger NETs formation, by engaging A1 and A3 adenosine receptors (ARs) and the induction of reactive oxygen species (ROS)– and peptidylarginine deaminase (PAD)–dependent pathways. M1 macrophages incubated with NETs derived from DADA2 patients released significantly greater amounts of TNF-α (66).

Further works are necessary to evaluate neutrophils’ role in DADA2 pathogenesis, possibly examining immune pathways activation with single cells technology, as already performed for other immune cells lines (39, 49) and their interactions with other cellular types.

### T Cells, Unconventional T Cells (NKT, γδT, MAIT) and B Cells: Only Supporting Actors or Deserve a Main Role?

ADA2 increases the rate of proliferation of monocyte-activated CD4+ T cells, acting as growth factor and induces T cell-dependent differentiation of monocytes into macrophages, stimulating macrophage proliferation (22, 23). Therefore, ADA2 deficiency impact on T cell compartment was not unexpected. Despite an initial report of normal T cells subsets (1), subsequent studies showed a reduction in helper and cytotoxic T subpopulations (12, 18, 55, 67) and a significant increase of cTfh cell frequency, likely secondary to the ongoing chronic inflammation (67). DADA2 cTfh cells present an impaired IL-21 production, a crucial cytokine for B-cell help function. A further alteration of T cells is linked to a reduced expression of CD40 ligand (CD40L), associated with impaired helper activity toward B-cells, indicating also an alteration in the T–B cell interaction process (67).

More recently, an impaired differentiation of CD4+ and CD8+ memory T cells *in vivo* has been highlighted (12, 67). CD4+ and CD8+ T cells in DADA2 patients display an exacerbated senescent/exhausted phenotype, as shown by cellular markers, such as increased CD95 and CD57, decreased CD28 and increased CX3CR1 (12). ADA2-deficient CD8+ TEM/TEMRA cells secrete lower levels of granzyme A (12), whereas cytokines secretion and effector function of CD4+ T cells seem unaffected. Furthermore, a significantly reduced proportion of Treg cells was recently identified in DADA2 patients (12) and lower proportions of unconventional NKT (12, 24) and mucosal-associated invariant T (MAIT) (12) were also evident.

These data, together with a remarkably lower proportion of Vγδ2+T cells (12), suggest that DADA2 CD8+ T cells may be involved in the development of refractory/recurrent viral infections observed in some patients (54, 68–70). The defect in ADA2-deficient cytotoxic lymphocytes may be also linked to rare DADA2-associated manifestations, such as hemophagocytic lymphohistiocytosis (HLH) (1, 5, 56) or CD3+ CD8+ large granular lymphocytes (11, 18).

An increased proportion of CD4-CD8- αß+ TCR+ cells is also reported in several patients (18–20, 71), consistently with an ALPS-like phenotype (3, 11, 71, 72).

Profiling of the TCR repertoires at the single-cell levels showed no significant clonal dominance of T cells, most TCRs were individual-private, and there were no disease-specific TCRs indicating as unlikely the possibility of a common pathogenic background (49, 73).

T-cells transcriptomic analysis in DADA2 revealed an activation of IFN pathways (both IFN*α*/*β* and IFN*γ* signaling**)** as a T cells signature and *STAT1* as a hub gene in the gene network of T cell activation and cytotoxicity (49). Notably, many functions of IFN*γ* have been ascribed to direct STAT1- mediated induction of immune effector genes. STAT1 activation could also be mediated by cross regulation of cellular responses to other cytokines and inflammatory factors (74).

As mentioned above, defective B cell function is linked to defective cTfh help towards B cells (67), but also an intrinsic defect of B cell lineage itself has been shown (12, 67).

ADA2 binds B cells (23), which have also the ability to produce and secrete ADA2 (67). The involvement of the B-cell compartment in DADA2 was evident since first reports. Zhou et al. (1) described a B cells defect in terminal maturation and a tendency towards apoptosis, consistent with a clinical phenotype characterized by hypogammaglobulinemia and recurrent infections together with the typical inflammatory features (18) (**Figure 1**).

The most striking effect of ADA2 deficiency on B cells is the impairment of their differentiation, which seems compromised at several levels. A reduction of memory B cells is confirmed by different studies (1, 2, 18, 27, 55, 67). Recent findings (12, 18, 67) show that ADA2-deficiency alters not only the generation and/or maintenance of the memory B cell pool, but also the ability of B cells to undergo Ig class switching, mainly to IgA secreting cells (12). In the study by Yap et al, the absolute levels of CD38 and IgM expressed were significantly higher on transitional, naïve and memory B cells from DADA2 patients than healthy donors (12). An increase in naive (12, 27, 55) and transitional B cells subsets (12, 18) has been also found. In conclusion, DADA2 B cells populations seem to be less mature than those of healthy controls (12), with an impairment in their ability to proliferate and differentiate. DADA2 naıve B-cells display also a reduced capacity to respond to TLR 9 agonist, CD40L, and anti-human Ig stimulation, suggesting an intrinsic proliferation defect induced by mutations of ADA2 (67). Furthermore, an expansion of CD21low B cells has been shown (67), possibly being a potential predisposing factor to autoimmunity (18, 75).

It has to be noted that, compared with HDs, BM from DADA2 patients may exhibit a reduced number of mature and immature populations belonging to different hematopoietic lineages, B lineage included (36). Notably, the aberrant distribution and phenotype of peripheral B cells subsets in DADA2 patients could be the consequence of a primary block of maturation of B precursors in the BM, as recently shown in one untreated patient, with an accumulation of pre-B cells and immature B cells (12).

## Discussion

Since the first studies on DADA2 (1, 2) it was clear that DADA2 pathogenesis and clinical heterogeneity could not be completely explained by macrophage involvement alone, possibly implicating different immune cell lineages participation (**Figure 2**). In fact, DADA2 is an atypical autoinflammatory disease, in which the causative gene is not involved in the canonical inflammatory pathways, but rather affects purines metabolism and signaling (24, 76, 77). Monocytes/macrophages remain central actors in the pathogenesis of vasculitis-related phenotype (37), but other mechanisms have been recently shown to contribute to endothelial disruption, such as increased NETosis and NETs stimulatory effect on macrophages itself (62).

Additional works unraveled alterations across all the immune system, possibly linked to the pleiotropic clinical features in DADA2 (**Figure 1**): an intrinsic defect of B cell compartment may be related not only to recurrent and chronic infections (12, 18, 27), but also to autoimmunity development (18, 48, 75), which can be additionally influenced by a T reg defect (12) and an hyperactivation of IFN type axis (12, 37, 44–48, 78).

Predisposition to viral infections may be justified by CD8+ alterations and Vγδ2+T cells defect (12) together with the increased proportion of NK regulatory cells (12). These alterations could be also implicated in the reported HLH episodes (1, 7, 56, 79).

Hematological manifestations have been described (7, 14, 17, 18, 38, 39, 43, 55, 60, 80, 81), but their pathophysiology is still mostly obscure. In addition to the maturation block of patients hematopoietic progenitors (HPC) (12, 36), patients’ marrow plasma (19, 71) showed an inhibitory effect on growth and differentiation of healthy BM precursors, possibly suggesting a humoral inhibitory effect. ADA2 may have a role in hematopoietic processes besides its inflammation-associated effects. Recently a genotype-phenotype correlation (13) demonstrated that mutations leading to complete (or almost) loss of ADA2 activity are associated with a prevalent hematological phenotype. We could hypothesize that the coexistence of inflammatory, immunodeficiency and hematologic phenotypes could be independent of ADA2 enzyme activity per se, because it is known that ADA2 protein binds to multiple immune cells (22, 23) and may exert different effects on different target cells, possibly acting as growth factor also for HPC.

Specific studies investigating DADA2 patients BM microenvironment and hematopoiesis regulation are mandatory to elucidate the possibility of an intrinsic defect in DADA2 HPC or extrinsic factors in BM niche (19, 71).

Definitely, so far few cytokines have shown a central role in DADA2: TNF-α and, lately, type I and type II IFN (82).

TNF-α is one of the main mediators of vascular inflammation (83) and several studies showed detectable levels of TNF-α in the peripheral blood and affected tissues from DADA2 patients with active disease (1, 9, 37, 39). Its biological effects include activation of other immune cells such as macrophages, DCs, T-cells and B-cells (84) and inhibition of Treg-cells, consistently with reduced Tregs number in DADA2 patients.

Notably, reduced ADA2 activity has been associated with intracellular accumulation of deoxyinosine, interfering with the cellular methionine cycle through the inhibition of SAM synthetase activity. This mechanism drives epigenomic hypomethylation and overexpression of immune-stimulatory endogenous retroviral elements, inducing IFN-β production (77).

How the activation of type I IFN in DADA2 patients contributes to the pathophysiology is still not completely clarified. It is known that IFNα/β enhance NK cytotoxicity, effector CD4+ and CD8+ T cell responses and B cell differentiation into plasma cells and antibody production, essential mechanisms leading to autoimmune response (78).

Recent studies show an activation of the IFN–JAK–STAT1 pathway in DADA2 patients (37, 43), mainly in monocytes (39) and T cells (49). The IFN-STAT1 axis could be an efficient inducer of M1 macrophages polarization, with a possible reciprocal cross-regulation of TNF and IFN production (73).

Interestingly, IFNγ and TNF-α have been described as possibly implicated in the pathophysiology of aplastic anemia (85) and abnormal STAT1 activation was demonstrated in BM samples of few aplastic anemia patients (86). Given the recent observation of STAT1 axis activation in DADA2 patients, its possible role in DADA2 hematological compartment defect should be explored.

At the moment, DADA2 treatment may differ based on clinical phenotype: most inflammatory features can be effectively controlled using TNF inhibitors, with reduction of stroke recurrence (18, 87, 88), improvement of blood vessels endothelial integrity and resolution of inflammatory myeloid cell infiltrates (37). Nevertheless, TNFi are not so effective at controlling hematological manifestations (13, 60, 61, 87, 89, 90). For these patients, hematopoietic stem cell transplantation has been reported reverting both hematological and inflammatory features and restoring ADA2 activity (15, 16, 18). Recent gene therapy preclinical studies demonstrate the correction of proinflammatory profile in patients’ macrophages after lentiviral-mediated ADA2 gene transfer (36, 38) and recovery of stem cell proliferation and colony forming units capacity in CD34+ HSPC (38). Further exploration of IFNs role in DADA2 pathophysiological process may open new therapeutic options for these patients, such as JAK-inhibitors or anti-IFNγ antibodies, as well as NETs inhibitors if NETosis involvement in DADA2 pathogenesis will be reinforced.

In conclusion, ADA2 deficiency has a complex impact on both innate and adaptive immunity cell lineages. Recent studies helped to start clarifying the pathophysiological mechanism underlying several clinical manifestations, but wide gaps still need to be filled. Future therapeutic approaches, such as gene therapy, will need to take into consideration the spread impact of ADA2 deficiency on the immune system.

## Author Contributions

FS and SS conceived the idea, reviewed the literature, and wrote the first draft of the manuscript. AB and FP created the figures. RC, MG, SV, AC, and GC critically reviewed the manuscript and edited the manuscript. All authors contributed to the article and approved the submitted version.

## Funding

This work was partially supported by the Italian Ministry of Health—Ricerca Finalizzata (grant RF-2019-12370600) to MG, and partially funded by the Italian Ministry of Health, RC2021.

## Conflict of Interest

The authors declare that the research was conducted in the absence of any commercial or financial relationships that could be construed as a potential conflict of interest.

## Publisher’s Note

All claims expressed in this article are solely those of the authors and do not necessarily represent those of their affiliated organizations, or those of the publisher, the editors and the reviewers. Any product that may be evaluated in this article, or claim that may be made by its manufacturer, is not guaranteed or endorsed by the publisher.

## Footnotes

1
https://www.proteinatlas.org/ENSG00000093072-ADA2/immune+cell, https://www.proteinatlas.org/ENSG00000093072-ADA2/single+cell+type.
